# Adhesion Performance in the Eggs of the Philippine Leaf Insect *Phyllium philippinicum* (Phasmatodea: Phylliidae)

**DOI:** 10.3390/insects11070400

**Published:** 2020-06-28

**Authors:** Thies H. Büscher, Elise Quigley, Stanislav N. Gorb

**Affiliations:** Department of Functional Morphology and Biomechanics, Institute of Zoology, Kiel University, Am Botanischen Garten 9, 24118 Kiel, Germany; eliseq2@scarletmail.rutgers.edu (E.Q.); sgorb@zoologie.uni-kiel.de (S.N.G.)

**Keywords:** attachment, glue, oviposition, biomechanics, walking leaf, morphology, plant surface interactions, insect–plant relations, egg dispersal

## Abstract

Leaf insects (Phasmatodea: Phylliidae) exhibit perfect crypsis imitating leaves. Although the special appearance of the eggs of the species *Phyllium philippinicum*, which imitate plant seeds, has received attention in different taxonomic studies, the attachment capability of the eggs remains rather anecdotical. We herein elucidate the specialized attachment mechanism of the eggs of this species and provide the first experimental approach to systematically characterize the functional properties of their adhesion by using different microscopy techniques and attachment force measurements on substrates with differing degrees of roughness and surface chemistry, as well as repetitive attachment/detachment cycles while under the influence of water contact. We found that a combination of folded exochorionic structures (pinnae) and a film of adhesive secretion contribute to attachment, which both respond to water. Adhesion is initiated by the glue, which becomes fluid through hydration, enabling adaption to the surface profile. Hierarchically structured pinnae support the spreading of the glue and reinforcement of the film. This combination aids the egg’s surface in adapting to the surface roughness, yet the attachment strength is additionally influenced by the egg’s surface chemistry, favoring hydrophilic substrates. Repetitive detachment and water-mediated adhesion can optimize the location of the egg to ensure suitable environmental conditions for embryonic development. Furthermore, this repeatable and water-controlled adhesion mechanism can stimulate further research for biomimeticists, ecologists and conservationalists.

## 1. Introduction

Stick insects (Phasmatodea) are rather large terrestrial herbivores and well known for their remarkable camouflage [1,2]. This masquerade, imitating parts of their environment, is particularly striking in the lineage Phylliidae (leaf insects). Consequently, these insects are commonly called “walking leaves” [3,4,5]. Leaf insects extraordinarily imitate the leaves of plants and visually merge with their environment. The first fossil records of Phylliidae date back 47 mya with *Eophyllium messelensis* Wedmann, Bradler and Rust 2006 as the oldest known representative of this lineage [6]. Visual camouflage in stick insects had already evolved during the Cretaceous period (approximately 125 mya), to avoid predators at a time when gymnosperm plants represented the majority of plant diversity [6,7,8]. During the emergence of angiosperms and their major radiation [9,10], stick insects evolved in a similar rapid fashion, possibly as a response to the burgeoning diversity of plants [8,11,12,13,14]. Camouflage is not only used by the insects to deceive predators, but also exhibited by the eggs in their resemblance to plant seeds [1,13,15]. Beyond visual aspects, ranging from the imitation of twigs, bark, moss and other environmental elements, along with the convergent evolution of leaf mimicry in Phyliidae and several other groups of stick insects [16], other characteristics diversified as well. The attachment systems of phasmids, for example, adapted to the abundance of different plant surfaces [5,17,18,19,20,21]. Females also made use of a remarkably broad range of oviposition techniques, which differ between species depending on their ecological niche [2,5]. As a result, the egg morphology reflects an oviposition technique and ecological niche [15,22,23]. Some species simply drop their eggs passively while others catapult them actively. Passively dropping the eggs is considered an extension of phasmids’ notorious masquerade crypsis [2] and probably an ancestral technique [2,13,24]. Another widespread principle is fixation of one egg or groups of eggs to specific spots, e.g., their host plants [13]. While some species mechanically drill their eggs into the soil, into crevices or even leaves and bark, other species secrete a glue during oviposition to permanently fix the eggs to the substrate [2,5,13,20]. The latter is either used to attach single eggs or batches to a certain place, and in one striking case, the egg batches are deposited in the form of an ootheca with a protective case [13]. The different strategies for egg deposition are a result of the low spatial distribution and extensive radiation of phasmids, which presumably led to the co-evolution with angiosperms. 

Interestingly, not only has the outer appearance of phasmids been shaped by their co-existence with plants, but also the eggs of phasmids mimic the appearance of seeds and even copy functional principles of seeds. Phasmids are not only the sole insect lineage with species-specific egg appearances [2], but also the only lineage with eggs adapted to different oviposition techniques. Some taxa, in which the eggs are dropped passively, produce eggs which bear a capitulum. This extension of the egg’s operculum is not only a signal adaptation for zoochory by ants, but also a result of co-evolution with plant seeds and ants [25,26]. In both capitulate phasmid eggs and elaiosome-bearing seeds, such a lipid-rich extension mimics ant-specific signaling and convinces ants to carry the egg or seed and thereby mediate dispersal [27,28,29]. Besides ant-mediated zoochory, the eggs of several species of phasmids follow the same principles that plant seeds deploy for dispersion, or aggregation respectively. Many plant seeds disperse via endozoochory, especially via birds [30,31]. Although an initial study has shown that phasmid eggs (directly fed to birds) of a few species do not survive the digestion by quills and ducks [32], a subsequent study found the eggs of several other phasmid species remain viable inside a gravid female phasmid that has been consumed by a bird [33]. Other phasmid eggs, especially *Megacrania* species, are experimentally shown to float in sea water, and disperse via the ocean [34,35,36,37], like the seeds of *Cycas* spp. (Cycadaceae) or screw pines do [38,39]. 

**Figure 1:**
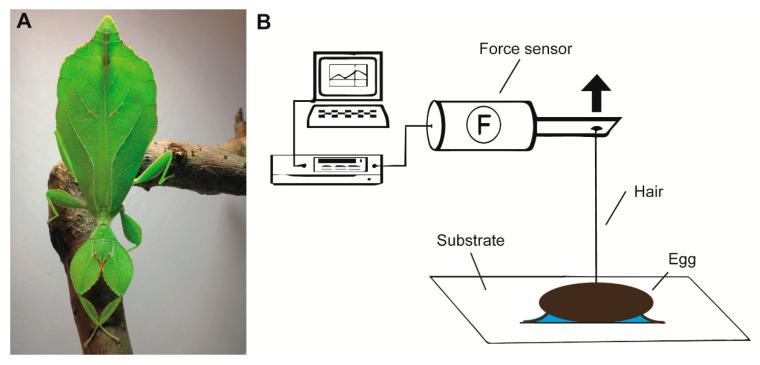
Examined species and experimental setup. (**A**) Female of *Phyllium philippinicum* (image is provided by Daniel Dittmar). (**B**) Experimental set-up for detachment force measurements. The egg, which was glued onto the particular substrate fixed on a lab boy using double-sided sticky tape. A hair was glued onto the egg and connected to a force sensor. To detach the egg from the substrate, the force sensor was moved away from the egg in perpendicular direction. The time force signal was amplified and finally processed in a computer.

*Phyllium philippinicum* Hennemann, Conle, Gottardo and Bresseel, 2009 (Phylliidae) is a species of leaf insect commonly bred in labs and private cultures (Figure 1A). However, most of the literature on the species revolves around taxonomic and phylogenetic classification and is mainly based on adult morphology [3,40,41,42]. Leaf insects in general are reported to drop or catapult their egg for deposition from the canopy tops of their host tree [2,13]. Basically, the eggs of this species, as well as those of closely related species, employ a more specialized mechanism for host plant association than previously reported. The specialized exochorionic morphology of leaf insect eggs is predominantly accounted in descriptive morphological studies and taxonomic descriptions [4,15,23,42] and, hence, functional aspects have been widely undocumented. The eggs of several *Phyllium* species, including *P. philippinicum*, resemble plant seeds and bear protruding exochorionic structures (pinnae, according to Clark [43]). The morphology of these pinnae is suggested to be species-specific and their taxonomic use has been previously well demonstrated [4,42]. The functionality of these structures is thus far largely unknown, but the unfolding behavior of the pinnae is often observed in captive breeding. The fact that the pinnae morphologically respond to water has anecdotally raised questions amongst the phasmid breeding community on what purpose this mechanism might serve. Only very few taxonomic studies hypothesized the function of these pinnae. Hennemann et al. [3] described the unfolding of the pinnae after their contact with water and suggested an adhesive function of this system, however did not further elucidate this idea. Additionally curious, the oviposition technique employed by the females, does not involve active gluing of the eggs, which begs the question of whether there is a presence or absence of accessory reproductive glands in *P. philippinicum*. Unfortunately, most studies on leaf insects solely focus on external morphological features, leaving this question unanswered.

Overall, strong egg attachment has been reported in a number of other insect species on natural substrates [44,45,46,47] and even stronger adhesion was measured from extracted egg glue on various artificial substrates [48,49,50]. The specific properties of the egg glue seemingly depend on the level of specialization in the attachment system and the habitat/substrate it is specialized for, therefore resulting in different strategies [51]. One important component influencing attachment efficiency is the roughness of the substrate: rougher surfaces create a greater contact area for glue and stronger adhesion after glue solidification [52]. The high complexity in the structural features of plant leaves (trichomes, wax crystals, stomata and cuticle foldings) and fruits (microcracks and epicuticular wax crystals) of various plant cultivars leads to rougher surfaces and increases the adhesion of the eggs of the codling moth *Cydia pomonella* (Linnaeus, 1758) (Lepidoptera, Tortricidae), as experimentally shown [44,45]. The egg attachment strength of the parasitic warble fly (Diptera, Hypoderminae) positively correlates to the roughness of the hairs on its host species [53]. Insect vectors of the human bot fly *Dermatobia hominis* (Linnaeus Jr., 1781) (Diptera, Oestridae) are covered with setae, which enhances egg adhesion for the human bot fly [54]. Another important factor influencing the attachment of eggs is surface chemistry. Eggs of the asparagus beetle *Crioceris asparagi* (Linnaeus, 1758) (Coleoptera, Chrysomelidae) adhere well to the surfaces of the plant *Asparagus officinalis* L. (Asparagaceae), which have superhydrophobic and microstructured surfaces due to the coating by wax crystals [46].

Eggs with adhesive responses in contact with water are only reported for a few insect species. The dragonfly *Libellula depressa* Linnaeus, 1758 (Odonata, Libellulidae), and other Anisoptera [55,56,57,58,59,60,61,62,63,64] lay eggs which possess an adhesive coating that swells and generates adhesive properties after the female deposits them in water [65]. The eggs of Ephemeroptera are covered with a thick layer composed of tightly entwined filaments, causing cohesion of the eggs and adhesion to a substrate after deposition into water [66]. The exochorionic structures of these species undergo modifications upon interaction with water, in turn generating adhesion [65,66]. It is assumed that in lieu of colleterial glands [55,56,67], these adhesive coatings are synthesized by follicle cells [65,68] which are involved in eggshell deposition [66,69,70,71].

On one hand, exploring the adhesive properties and response to water contact of the eggs of *P. philippinicum* can enhance our knowledge of multifunctional bioadhesives. On the other hand, this functional system can provide insights into the life history of this species and shed light on the ecological environments this species inhabits, as this knowledge is usually missing in taxonomic descriptions of museum specimens. This could assist future studies in obtaining broader ecological knowledge of this species, contributing to conservational aspects for both phasmids and plants that can be subject to damage by insects, and also give input on evolutionary studies, as the highly specialized attachment mechanism of *P. philippinicum* is highly derived. In this paper, we asked the following specific questions. (i) How do the eggs of *P. philippinicum* adhere? (ii) How do water contact, surface topography and surface chemistry influence egg adhesion in this species? (iii) Is attachment in *P. philippinicum* eggs reversible and repeatable? 

## 2. Materials and Methods

### 2.1. Specimens

The eggs of *Phyllium philippinicum* Hennemann, Conle, Gottardo and Bresseel, 2009 were obtained shortly after being laid by female insects from the culture of Kirsten Weibert (Jena, Germany). The animals were fed with blackberry leaves ad libitum and kept in a natural day/night cycle. The weight of freshly laid eggs (*N* = 20) was measured using an analytical balance AG204 Delta Range microbalance (Mettler Toledo, Greifensee, Switzerland; d = 0.1 mg).

### 2.2. Morphology

Eggs attached to microscopy glass slides were observed with the Leica Microscope M205 (Leica Microsystems Ltd., Wetzlar, Germany). Images were captured from both sides, overview of the egg and view of the contact through the glass slide, using the microscope camera Leica DFC420 (Leica Microsystems Ltd., Wetzlar, Germany). Multifocus stacked images were postprocessed using the software Leica Application Suite (LAS) version 3.8.0 (Leica Microsystems Ltd., Wetzlar, Germany) and Affinity Photo (Apple Inc., Cupertino, CA, USA).

For higher magnification, eggs in contact with different substrates, as well as detached and untreated eggs, were air-dried and sputter-coated with gold-palladium of 10 nm thickness. The substrates corresponding to the detached eggs were sputter-coated as well. Additionally, some untreated eggs were dehydrated using an ascending alcohol series, critical point-dried and sputter-coated as well. These samples were observed in the SEM Hitachi S4800 (Hitachi High-technologies Corp., Tokio, Japan) at an acceleration voltage of 5 kV. Subsequently, the images were processed with Affinity Photo (Apple Inc., Cupertino, CA, USA).

The nomenclature of the egg morphology follows Sellick [23].

### 2.3. Detachment Force Measurements

**Figure 2:**
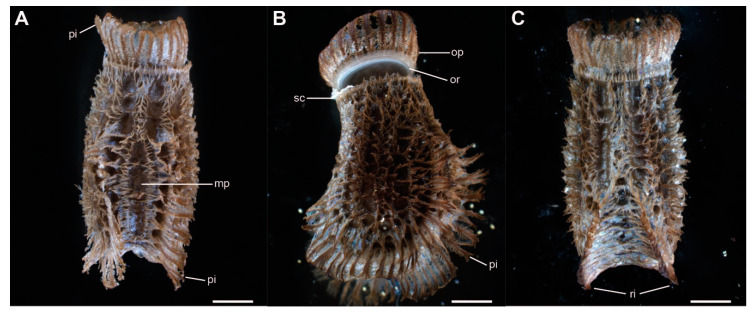
Morphology of the eggs of *Phyllium philippinicum*. (**A**) Dorsal view. (**B**) Lateral view. (**C**) Ventral view. **mp**, micropyle; **op**, operculum; **or**, opercular rim; **pi**, pinnae; **ri**, ribs; **sc**, serosal cuticle. Scale bars: 1 mm.

The detachment force of individual eggs was measured in four different experiments. In all experiments, the eggs were mounted on standardized surfaces, as described below, and individually attached to a force transducer (100 g capacity; FORT100, World Precision Instruments Inc., Sarasota, FL, USA) by gluing a horsehair with bees wax onto the lateral side of the egg (Figure 2B) and attaching the hair to the sensor (Figure 1B). The force transducer was connected to a BIOPAC Model MP100 and a BIOPAC TCI-102 system (BIOPAC Systems, Inc., Goleta, CA, USA). Force–time curves were recorded by pulling the eggs off the surfaces using the software Acqknowledge 3.7.0 (BIOPAC Systems Inc., Goleta, CA, USA). The test surfaces were lowered away from the sensor with a speed of approximately 2–3 cm/s using a laboratory lifting platform. In all four experiments, the detachment force was measured by pulling the egg off of a surface at an angle of 90°, with the same setup, as described by Wohlfart et al. [72] for spiders and later used for adult stick insects [19]. The highest peak of the visualized graph was interpreted as the maximum detachment force. All surfaces were carefully cleaned with 70% isopropylic alcohol prior to each experiment. Detachment forces were measured in the following four different experiments: 

(1) Freshly laid eggs (*N* = 32 per substrate) were mounted on four test substrates with different roughness (0, 1 and 12 µm, and standardized p40 polishing paper) made of epoxy resin (as described below). Eggs were prepared on the test substrates by placing individual droplets of distilled water (~100 μL) on the epoxide plates and then placing one egg in a single droplet, to trigger the unfolding of the pinnae. Subsequently, the eggs were allowed to dry completely (~24 h) and then attached to the sensor.

(2) Eggs (*N* = 20 per substrate) were mounted on three surfaces with different chemical surface properties with the same procedure as described above. The surfaces used differed in the wettability, indicated by the contact angle of the water, which was 36.25 ± 1.15° (mean ± SD, *n* = 10) (hydrophilic), 83.38 ± 0.89° (the same epoxy resin as used for experiment 1) and 98.9 ± 0.47° (hydrophobic). 

(3) Additionally, eggs were placed on the hydrophobic and the hydrophilic substrates in wet condition (*N* = 20 per substrate) and the detachment force was measured. The eggs were individually fastened with a horsehair as described above and fully submerged in distilled water for 20 min; afterwards, they were attached to the force transducer and then placed on the test substrate. After letting the eggs sit on the substrate for 1 min, the detachment force from the substrate was measured in the same manner as in the other experiments. 

(4) The reproducibility of egg attachment was tested by subsequent pull-off measurements of the same egg. Individual eggs (*N* = 8) were prepared as described in the first experiment and attached to the smooth epoxy resin substrate (0 µm roughness). Then, the detachment force was measured by pulling off the egg. Afterwards, the same egg was then reattached once again using a droplet of water and left to dry for another 24 h. This procedure was repeated for each of the eight eggs six different times, until the measured detachment forces were similar in comparison to the previous day (i.e., revealed no significant difference).

All experiments were performed at 19–21 °C temperature and 45–55% relative humidity.

### 2.4. Surface Preparation

Two different types of surfaces were used in the experiments. Epoxy resin with a different surface roughness for the first and the fourth experiment and glass with different wettability, as well as epoxy resin, for the second and third experiments.

#### 2.4.1. Glass

Clean microscope glass slides (Carl Roth GmbH & Co. KG, Karlsruhe, Germany) were used as the hydrophilic substrate and silanized, as described by Voigt and Gorb [46], to obtain a hydrophobic substrate. The surface chemistry was characterized by measuring the contact angle of the water on the substrate (aqua Millipore, droplet size = 1 μL, sessile drop method; *n* = 10 per substrate) using the contact angle measurement instrument OCAH 200 (Dataphysics Instruments GmbH, Filderstadt, Germany). The contact angle of the water was 36.25 ± 1.15° for the hydrophilic glass substrate and 98.9 ± 0.47° for the hydrophobic one.

#### 2.4.2. Epoxy Resin

Substrates with different roughness were produced using epoxy resin [73] following the protocol of Salerno et al. [74]. Negative replicas were cast using polyvinylsiloxane (PVS)-based two-component dental wax (Colthéne/Whaledent AG, Altstatten, Switzerland). Negatives were then filled with epoxy resin and cured at 70 °C for 24 h. Glass (0 µm roughness) and polishing papers with the roughness of 1 μm, 12 μm (Buehler, Lake Bluff, IL, USA) and industrially standardized p40 polishing paper (particle size ~440 µm) were used as templates for the resin replicas. The contact angle of the water on the smooth epoxy resin was 83.38 ± 0.89° (mean ± SD, *n* = 10).

### 2.5. Statistical Analysis

Statistical analyses were performed with SigmaPlot 12.0 (Systat Software Inc., San José, CA, USA). Normal distribution and homoscedasticity were tested using the Shapiro–Wilk test and Levene’s test, respectively, prior to other tests. As the respective data were neither parametric nor showed homoscedasticity, detachment forces of eggs on substrates with different surface roughness, as well as on surfaces with different chemical properties represented by corresponding contact angles, were compared using Kruskal–Wallis one-way analyses of variance (ANOVA) on ranks followed by Tukey’s post hoc test. Detachment forces of wet and dry eggs on surfaces with different contact angles were compared using Kruskal–Wallis one-way ANOVA and Tukey’s test as well. The Mann–Whitney rank sum test was used to compare the detachment forces of eggs in the wet condition on hydrophilic and hydrophobic surfaces. For a comparison of the detachment forces over a six-day period of repeated measurements, a Friedman repeated measures ANOVA was performed along with a Tukey’s post hoc test.

## 3. Results

### 3.1. Egg Morphology

**Figure 3:**
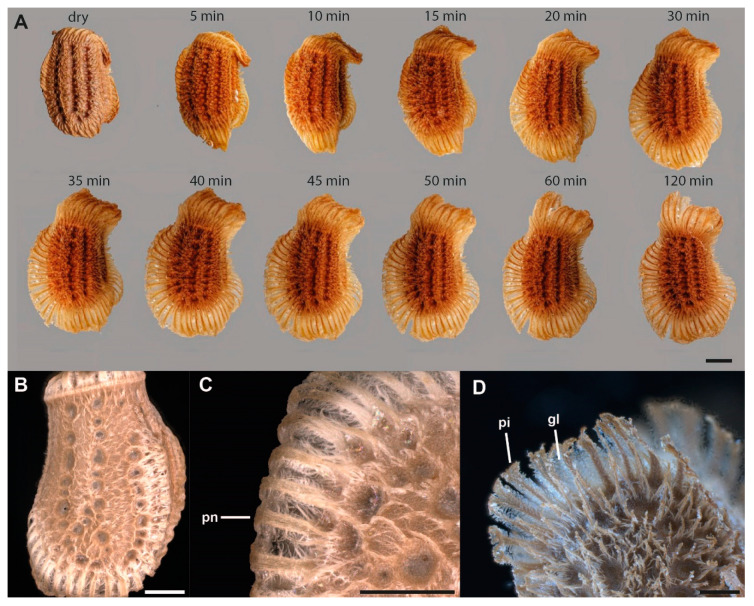
Unfolding behavior of *Phyllium* egg pinnae. (**A**) Succession of pinnae unfolding in *Phyllium rubrum* Cumming, Le Tirant and Teemsma, 2018, after exposure to water (images are provided by Bruno Kneubühler), lateral views. *B,C.* Lateral view of untreated *Phyllium philippinicum* egg. (**B**) Overview. (**C**) Detail of folded pinnae. (**D**) Detail of unfolded pinnae of a *Phyllium philippinicum* egg after water exposure. **gl**, glue; **pi**, unfolded pinna; **pn**, folded pinna. Scale bars: 1 mm (**A**,**B**), 500 µm (**C**,**D**).

The eggs of *Phyllium philippinicum* are laterally compressed and densely covered with small exochorionic appendages (pinnae, sensu Clark [43]). These pinnae cover most of the egg’s surface, except for some circular pits and the center of the micropylar plate (Figure 2A). A corona of shorter expansion surrounds the micropylar plate, oriented away from it. The anterior pole of the egg is covered by an operculum, the lid of the egg, which is released during the hatching of the nymph (Figure 2B). A formation of larger pinnae surrounds the outer rim of the operculum anteriorly. Two ribs along the lateral ridges of the egg are covered with long pinnae as well, expanding the lateral dimensions of the egg (Figure 2). The ribs meet on the ventral side of the egg (Figure 2C). Pinnae of freshly laid eggs lie flat on the surface of the egg, but unfold after contact with water, as described below (Figure 3). Dimensions of the eggs are measured according to Sellick [23]. They measure 4.39 ± 0.36 mm (mean ± SD, *N* = 7) in length, with a height of 2.77 ± 0.25 mm, and width of 2.16 ± 0.14 mm. The mean weight was 15.9 ± 1.3 mg (*N* = 20).

### 3.2. Pinnae Behavior and Adhesive Secretion

**Figure 4:**
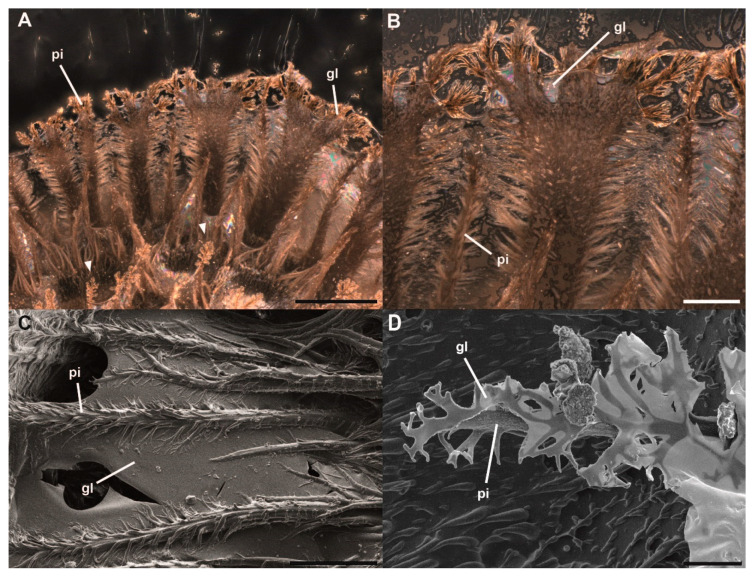
Glue associated with *Phyllium philippinicum* pinnae. (**A**,**B**) Stereomicroscopic images of pinnae attached to a glass surface, view through the glass slide. (**A**) Glue deposition on a glass surface and pinnae interaction with the substrate (arrowheads). (**B**). Reinforcement and distribution of the glue by the pinnae. (**C**,**D**) Scanning electron microscopy images of glue–pinnae interactions. *C.* Glue film adhering to pinnae. (**D**) Dried glue residuals on a pinna after detachment from a smooth glass surface. **gl**, glue; **pi**, pinnae. Scale bars: 500 µm (**A**), 300 µm (**B**), 100 µm (**C**), 10 µm (**D**).

The eggs are deposited by the female with the pinnae folded on the surface of the egg. A single pinna consists of a central shaft that is hierarchically split several times towards the tip (Figure 3D, 4). After oviposition, before initial contact with water, the folded pinnae are covered with an iridescent layer of a solidified secretion deposited by the female (Figure 3B,C). The pinnae unfold after contact with water and the secretion liquefies (Figure 3A). The larger pinnae on the operculum and the lateral ribs of the egg unroll and expand the dimension of the projected lateral area of the egg. Smaller pinnae, as well as hierarchical expansions of the main fringes of the pinnae, expand and increase the egg surface as well. The liquefied secretion on the surface of the eggs, after expansion of the pinnae, concentrates on the tips of the expansions (Figure 3D). Along the length of larger pinnae, a reservoir of the secretion forms a bridging film between the shafts of the pinnae. During contact with a substrate, the pinnae deform and spread the viscous secretion, in which they are imbedded, onto the substrate. After some time without contact to water (5–6 h), the secretion dehydrates and solidifies again (Figure 4A,B). After the curing off the secretion, the egg remains attached to the substrate. The adhesive function of the glue is characterized below.

### 3.3. Egg Attachment

**Figure 5:**
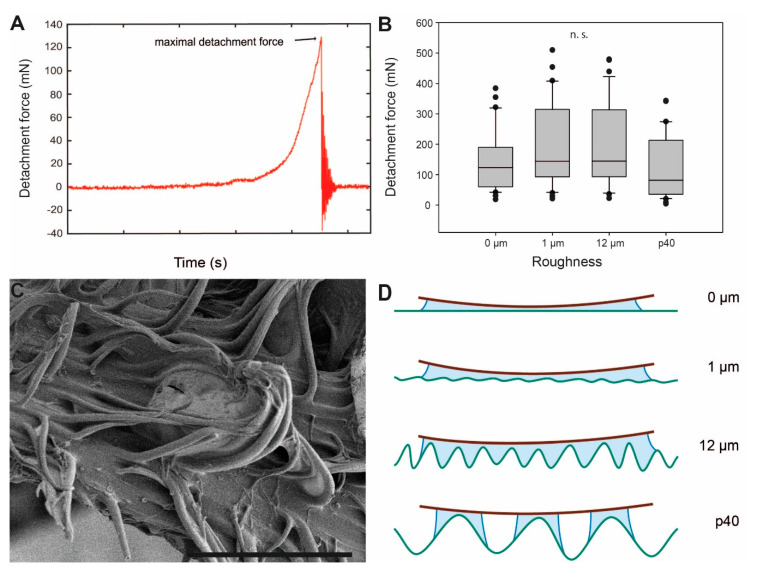
Influence of roughness on egg adhesion. (**A**). Exemplary force-time curve from measurements of the detachment force. (**B**) Detachment forces from substrates with different surface asperity (*N* = 32 for each roughness). Boxes are 25th and 75th percentiles, the line within the boxes defines the median, and whiskers represent the 10th and 90th percentiles. **n. s.** = no statistical difference (*p* > 0.05, Kruskal–Wallis ANOVA on ranks). (**C**) Scanning electron microscopy image of pinnae deformation showing the adaptation of pinna extensions to surface corrugations. (**D**) Schematic interpretation of the eggs’ glue with differing degrees of surface roughness. Roughness parameters are given in detail by Salerno et al. [74]. Scale bar: 60 µm.

The attachment performance of *P. philippinicum* eggs on different surface roughnesses is illustrated in Figure 5. The maximum pull-off force measured before the egg detached from the respective substrate (maximum detachment force, Figure 5A) is considered a measure for the attachment capability of the egg to the substrate. The maximum detachment force values were highest on the intermediate roughnesses, 12 μm with 144.65 ± 133.38 mN (median ± SD) and 1 µm with 144.23 ± 137.18 mN. The lowest detachment forces were recorded on the roughest (p40; 81.71 ± 104.11 mN) and the smoothest (0 µm; 122.94 ± 95.28 mN) surfaces. However, the differences in median detachment force values between the four surface roughnesses were not significant (Kruskal–Wallis one-way analysis of variance (ANOVA), *H* = 7.278, d.f. = 3, *p* = 0.064, *N* = 32 per roughness). 

**Figure 6:**
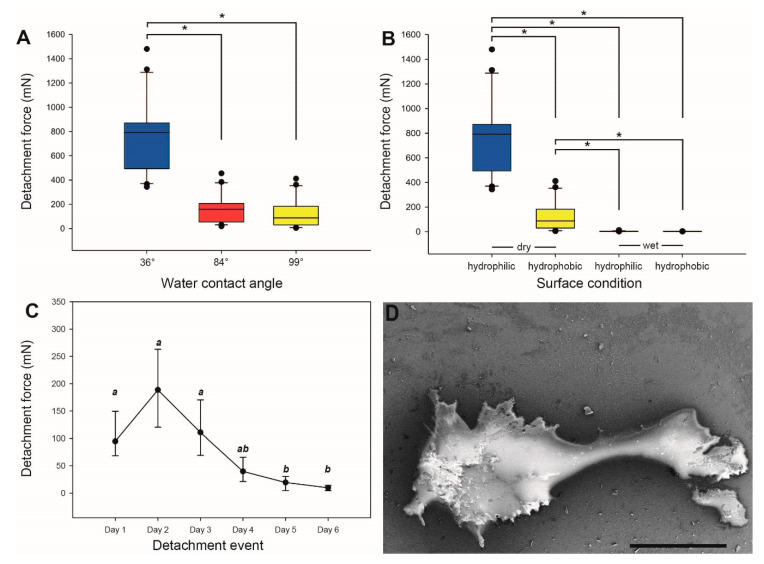
Influence of surface chemistry and repetitive detachment on *Phyllium philippinicum* eggs. (**A**) Detachment forces from surfaces with different water contact angles (*N* = 20 for each contact angle). (**B**) Detachment forces from wet and dry surfaces with different chemical properties (*N* = 20 for each treatment). “Hydrophilic” corresponds to a contact angle of 36° and “hydrophobic” corresponds to a 99° water contact angle. Boxes are 25th and 75th percentiles, the line within the boxes defines the median, and whiskers represent the 10th and 90th percentiles. * *p* ≤ 0.001, only significant comparisons are highlighted (Kruskal–Wallis one-way ANOVA on ranks followed by Tukey’s post hoc test). (**C**) Detachment forces during sequential detachment events (*N* = 8 eggs). Dots indicate the median, whiskers represent the standard deviation. Lowercase letters indicate statistically similarity. Groups with the same letter are statistically equal (Friedman repeated measurements ANOVA, followed by Tukey’s test). (**D**) Scanning electron microscopy image of glue residuals on a smooth hydrophobic glass surface. Scale bar: 300 µm.

The attachment performance of eggs on surfaces of differing surface chemistry is displayed in Figure 6A. The detachment force from pulling the eggs off of the hydrophilic surface (water contact angle 36.25°) was very high (792.37 ± 293.94 mN) and significantly higher than the force measured on surfaces with a higher water contact angle (Kruskal–Wallis one-way ANOVA, *H* = 38.543, d.f. = 2, *p* ≤ 0.001, *N* = 20 per surface; Tukey’s test, *p* < 0.05). The adhesion to epoxy resin (water contact angle 83.38°) was significantly lower than that of the hydrophilic glass with 159.03 ± 117.31 mN (Tukey’s test, *p* < 0.05), but higher than the adhesion to the hydrophobic glass (water contact angle 98.9 °) with 88.03 ± 114.81 mN. The latter difference, between the epoxy resin and hydrophobic silanized glass, was not found to be statistically significant according to Tukey’s post hoc test (Tukey’s test, *p* > 0.05). 

Adhesion to both hydrophobic and hydrophilic surfaces was very low and practically negligible in the presence of water on the surface (Figure 6B). Wet eggs on the hydrophobic surface (2.74 ± 0.34 mN) showed no significant difference in detachment values compared with wet eggs on the hydrophilic surface (3.17 ± 2.30 mN; Kruskal–Wallis one-way ANOVA, *H* = 66.77, d.f. = 3, *p* ≤ 0.001, *N* = 20 per treatment; Tukey’s post hoc test, *p* > 0.05). The comparison of the egg adhesion performance between hydrophobic and hydrophilic surfaces in both wet and dry conditions yielded significant differences between all comparisons, except for the comparison between the two wet surfaces (*p* < 0.05, Tukey’s test). Eggs dried in adherence to hydrophilic surfaces showed significantly higher detachment forces than eggs in contact with wet surfaces, as well as higher adhesion than eggs dried on hydrophobic surfaces (all *p* < 0.05, Tukey’s test). The detachment force of dried eggs from the hydrophobic glass was lower than from the hydrophilic substrates, but higher than wet eggs from both substrates (all *p* < 0.05, Tukey’s test).

Figure 6C illustrates the attachment performance of eggs over a sequence of six repeated detachment events. The median detachment force initially increased from day 1 (94.8 ± 38.0 mN) to day 2 (188.68 ± 66.75 mN). Subsequently, the detachment force consistently decreased from day 2 until day 6 (9.79 ± 4.56 mN). The detachment force was statistically different (Friedman repeated measures ANOVA on ranks, *χ*² = 35.179, d.f. = 5, *p* ≤ 0.001, *N* = 8 per day) and decreased between day 2 and day 6. However, the first three days were statistically similar, but each of the days 1–3 were significantly higher than days 5 and 6 (all *p* < 0.05, Tukey’s test). Although the overall decrease in attachment performance is significant, the initially higher median detachment force on day 2 is not significantly different from day 1 and day 3 (*p* > 0.05, Tukey’s test).

## 4. Discussion

### 4.1. Attachment Mechanism

The attachment capabilities of the eggs of *Phyllium philippinicum* were not readily paid attention to in the past and recognized only anecdotally in the literature [3]. However, the combination of an adhesive secretion and reinforcing microstructured exochorionic structures has proven to provide excellent attachment. The mean safety factor (*F_a_/F_m_*; mean detachment force per weight force) of eggs on a smooth epoxy resin substrate ranges around 924, i.e., the adhesion of one egg sufficiently attaches 924 times its own weight. On hydrophilic substrates, the average *F_a_/F_m_* is 4825. 

Water exposure has two main effects on the egg: (1) unfolding of the pinnae and (2) liquefaction of the glue (Figure 3). Both effects contribute to the enhancement of the adhesive properties of the eggs. Like a solvent-based adhesive, the egg adhesive dissolves partially in water and once the water evaporates, the adhesive dries and hardens on the substrate. When introduced to water, the pinnae extend and fan out, adapting to the texture of the substrate. The liquid glue covers the pinnae, which transmit and spread it out onto the substrate. Such horizontally oriented fibrillary structures, that lay parallel to a surface, facilitate the spreading of a fluid, hence enhancing the surface contact of the adhesive fluid [75]. Therefore, bridges of dried adhesive material between adjacent pinnae are visible, when re-solidified (Figure 3D, 4C). To achieve proper attachment, the glue becomes fluid to interact with an adjoining surface, then the adhesive fluid dries to either create a sufficient contact area [76,77] at the interface or mechanically interlock with the surface irregularities [77,78]. Whether high humidity in the surroundings or solely the contact to water droplets cause the glue to liquefy remains untested. To evaluate the effect of ambient humidity, further experiments with exposure to differing humidity are necessary. While the pinnae facilitate adhesion through an increased contact area with the surface, the fluid adhesive makes large real contact with the surface. The exochorionic extensions may also be able to extend into and interdigitate with surface asperities and further spread the adhesive fluid [79,80,81], depending on the roughness profile of the surface (Figure 5C). Their hierarchical structure offers finer subcontacts with the substrate [82,83], and, hence, optimizes contact formation on natural surfaces of fractal roughness with overlapping wavelengths (e.g., tree bark) [84]. Overall, the pinnae reinforce the film of the glue, thus achieving a viable adhesive system: soft enough to form intimate contact, yet stiff enough after solidification, to decrease elastic deformation and hold a strong bond [84]. This pinnae-based reinforcement offers structural integrity to the adhesive system of the egg.

Besides the mechanical interlocking of the solidified glue with surface corrugations, the glue adheres by physiochemical interactions, presumably van der Waals that can prove very strong with sufficient interfacial contact [77].

### 4.2. Influence of Substrate Roughness

Attachment on substrates with different surface roughness revealed no significant differences among all tested surfaces (Figure 5B). Other biological attachment systems are found to be significantly affected by surface roughness. The tarsal attachment systems of some flies and beetles consist of tenent setae that, similar to the pinnae of *P. philippinicum* eggs, adapt to the surface profile [85,86,87,88,89,90,91]. However, the performance of these attachment systems for the purpose of locomotion fare better on smooth surfaces or rougher surfaces exceeding asperity sizes of 3 µm, with the worst performance on micro-roughnesses ranging from 0.1–0.3 µm. This is explained by the spatula-like terminal elements of insects’ tenent setae interaction with the surface [85,89,90]. These setae tips are able to make sufficient contact with large surface asperities but are confounded by micro-rough surfaces that inhibit real contact of the setae to the surface. The eggs of *P. philippicum,* in contrast, performed well on all surface roughnesses tested.

This ability of *P. philippicum* eggs is presumably based on the action of initially fluid and later solidified glue. For glues, like that of the adhesive material of the eggs examined herein, rougher surfaces create a larger contact area and stronger adhesion [52]. This applies to the performance of *P. philippinicum* eggs, with an increasing trend in attachment strength from 0–12 µm roughness. Adhesion relies on the area of actual contact made with a surface [79,80,81]. Although surfaces with micro-roughness are not generally favourable for many insect attachment systems associated with locomotion, the egg’s adhesive fluid is able to conform around small surface irregularities and hence increase the actual contact area (Figure 5D). Rough surfaces are beneficial for egg adhesion in different insect species. For the codling moth, it has been previously shown that smoother surfaces with fewer trichomes and rather low free surface energy deter their eggs’ attachment [44], while structural features creating a rougher surface on leaves or fruits (e.g., trichomes, microcracks or epicutilar wax crystals) lead to stronger attachment of codling moth eggs due to an increase in the contact area with the egg’s glue [45]. Rough surfaces on plants are known to be favorable choices for oviposition sites in other lepidopterans [92,93] and the willow leaf beetle *Phratora vulgatissima* (Linnaeus, 1758) (Coleoptera, Chrysomelidae) [94], leading to an enhanced attachment strength. The same applies for the surface texture of the oviposition substrates of parasitic flies [53,54]. In contrast, the eggs of *P. philippinicum* adhere similarly strong to surfaces of all tested roughnesses. As the eggs are dropped without direct oviposition onto specific substrates, this case of universal adhesion is most likely adapted to a broad spectrum of surface roughness. 

However, p40, the roughest surface tested, revealed the weakest overall attachment to the eggs. The adhesive fluid probably generates a larger contact area on substrates with micro-rough surface corrugations. The large surface asperities of the p40 substrate (~440 µm asperity size) principally offer a larger surface for contact formation, but the glue of the egg presumably does not fill the deeper asperities, creating only partial interaction with the walls of the surface asperities (Figure 5D). The viscosity of the glue presumably prevents the glue from properly filling the surface texture.

### 4.3. Influence of Surface Chemistry

The adhesive strengths on surfaces with different water contact angles revealed significant differences between the attachment strength of eggs on the hydrophilic substrate compared with the two substrates with higher water contact angles. The contact formation and generation of attachment by the adhesive fluid depends on the surfaces’ chemical properties. Higher free surface energy and lower contact angles, which essentially vary inversely to one another [95], are characteristics of surface hydrophilicity that invite greater wetting of liquids on such a surface, in turn forming greater contact at the liquid–surface interface, which creates stronger adhesion [82,96,97]. Lower surface energy reduces the overall attachment ability of a system [84] and therefore a lower surface energy results in a lower detachment force of the eggs tested on the hydrophobic substrate. The same correlation between surface chemistry and attachment is reported for the tarsal attachment system of several groups of insects [87,88,98,99]. The water-mobilized adhesive presumably does not wet hydrophobic surfaces properly and therefore attachment is reduced, as wetting is an important prerequisite for this type of adhesion [78,100]. The adhesive fluid and its composition presumably best perform on hydrophilic surfaces, most likely due to polarity within the adhesive fluid and water that is attracted to polar/hydrophilic surfaces [78].

The range of suitable surface chemistry regimes in insect attachment is presumably a result of co-evolution of the insects and their corresponding plants [95]. Adaptation to substrate chemistry is species-specific and depends on the degrees of specialization to various natural substrates. Unfortunately, not many aspects of the ecology and natural habitat of *P. philippinicum* are known. Therefore, assumptions about their host trees are based on diet compatibility with certain leaves from their endemic region [3] and known host species of closely related *Phyllium* [1,101]. As *P. philippinicum* are generalist phytophages, there are several potential host plants. Some of the supposed host species of *P. philippinicum* are *Psidium guajava* L. (Myrtaceae), *Mangifera indica* L. (Anacardiaceae) and *Nephelium lappaceum* L. (Sapindaceae) [1,3,101]. The higher attachment performance on hydrophilic surfaces does not allow to specifically determine the actual host plants but enables an approximation of natural substrates the eggs are adapted to. All three putative food plants are evergreen tree species which have leaves that are generally hydrophobic with water contact angles around 100° [102]. In contrast, the bark of guava and mango was estimated to have contact angles around 52° and 50° [103]. The eggs more likely adhere to the bark than to the leaves. This is also reflected in the coloration of the eggs and freshly hatched nymphs. Nymphs hatch a dark brown hue and change to green after feeding on the foliage of the tree. Further tests are certainly necessary to test this assumption, but based on the findings herein, *P. philippinicum*’s eggs have a better chance at attaching to wood surfaces of its host tree species compared with leaf surfaces. Additionally, anchorage of the egg to a substrate could help the new-born nymph successfully hatch from the inside of the egg while remaining arboreal.

Apparently, for the eggs of *P. philippinicum*, the surface chemistry influences egg adhesion more than surface roughness. In experiments with beetles, their tarsal attachment systems worked the other way around. The hairy, wet adhesive pads of *Coccinella septempunctata* Linnaeus, 1758 (Coleoptera: Coccinellidae) and *Leptinotarsa decemlineata* Say, 1824 (Coleoptera: Chrysomelidae) responded more to surface roughness than to surface chemistry [51,104]. However, *Gastrophysa. viridula* (De Geer, 1775) (Coleoptera: Chrysomelidae) exhibited a decreasing attachment performance with hydrophobicity and stronger performance on smooth surfaces [87]. On the other hand, the eggs of *P. philippinicum* show decreased attachment on hydrophobic surfaces, but attachment values were high for all surface roughnesses. However, for attachment of the eggs, in contrast to moving insects, the preconditions are different. 

Two studies on egg adhesion of the codling moth used various natural surfaces with specific topographies and physiochemistries [44,45], however, the glue of the codling moth is permanent and most likely water-insoluble, and the adhesive of *P. philippinicum* is reversible and mostly water-soluble. Furthermore, these studies also found an effect of surface topography, which is probably due to the specific topography of the natural surfaces. Regardless, further investigation into the combined effect of surface chemistry and topography using a wider scope for both would be advantageous for the comparative effect on the attachment of *P. philippinicum* eggs.

### 4.4. Glue Properties

Detachment force values from *P. philippinicum* eggs in the wet condition were extremely low and practically negligible in comparison with other force measurements. It is assumed that the low force values in the wet condition reflects surface tension and capillary forces exerted by the water droplet that the egg was submerged in upon detachment, not the eggs’ actual attachment to the surface. 

Many insect eggs require hydration to become adhesive [55,57,58,59,60,61,62,63,64,65,66]. For example, eggs of the mayfly *Siphlonurus lacustris* (Eaton, 1870) (Ephemeroptera: Siphlonuridae) have a thick fibrous coat surrounding their eggs that undergo exochorionic changes once deposited in water, creating cohesion between egg masses and adhesion to a substrate [66]. However, these eggs attach in the wet condition and remain underwater. Contrastingly, the eggs examined herein need to dry after hydration to generate adhesion. The egg of *P. philippinicum* does not achieve attachment in water and necessitates a phase change from liquid to solid for the adhesive material to adhere. This could serve as a mechanism for the optimum site selection for the incubation of the egg to avoid adhesion under water, as attachment under water would be lethal to the hatching nymph. The eggs of *P. philippinicum* may be almost immediately ready to attach to a substrate once produced from the mother due to high humidity and the prevalence of water in a tropical rainforest [105]. This makes sufficient hydration of the egg highly probable and triggers its adhesive capabilities. However, if suitable conditions are not found, the egg retains the potential to adhere in a suitable environment in the next attachment event.

The glue mediated attachment of *P. philippinicum* eggs is reversible and reproducible over several cycles of attachment, detachment and reattachment (Figure 6C). Apparently, no glue is secreted by the egg itself; furthermore, colleterial glands for glue production are probably absent in the females as well [106], similar to some odonates and mayflies [65,66]. The fact that the attachment strength decreases after a few cycles of reattachment is probably due to the depletion in the supply of the glue covering the egg. The test surfaces revealed residuals of the glue after detachment (Figure 6D). However, the repeated ability to attach after submersion in water shows that the adhesive material is not entirely water-soluble and most of the secretion remains on the egg. In all likelihood, a hydrophilic polar portion of the material allows diffusion in water, facilitating adsorption at the glue interface [107] and consequently facilitates contact adaptation to the substrate [78]. A hydrophobic nonpolar portion most likely remains on the egg, preventing full dissolution [108]. The oviposition method that *P. philippinicum* females employ does not give them as much control over the oviposition site compared with other species that directly deposit eggs. The reversibility of adhesion may be a technique to correct maladaptive attachment sites or to adapt to seasonal changes in the environment, as reported for the egg glue of some alpine butterflies [47]. 

The chemical composition of the glue remains ambiguous and is not the subject of this study. However, some assumptions can be drawn based on the experimental results. Most permanent bioadhesives involved in egg attachment are largely proteinaceous [48,109,110,111,112,113,114,115]. The amphiphilic nature of the glue could be achieved by glycoproteins, as in many other insect glues [116]. The highly soluble glycan would serve as the polar portion [48,114,117], facilitating non-covalent bonding with hydrophilic substrates. The protein serves as the hydrophobic portion [108,118], providing adherence of the glue to the surface of the egg and its appendages. 

## 5. Conclusions

Although the special appearance of the eggs of *Phyllium* species, including *P. philippinicum*, received attention in different taxonomic and evolutionary studies [15,23,42], only a few hypotheses on the function of the special morphological features were presented [3]. We herein elucidate the specialized attachment mechanism of the eggs of this species and provide the first experimental approach to systematically characterize the functional properties of their adhesion. The adhesive mechanism of the egg exploits a combination of folded exochorionic structures (pinnae) and a film of adhesive secretion. Both components respond to contact with water. The glue becomes fluid through hydration, adapts to the substrate profile and adheres after solidification. The pinnae facilitate the spreading of the glue, support adaptability using hierarchically splitting filaments and reinforce the hardened film. This mechanism copes with surface roughness using this combination but is affected by surface chemistry. The glue adheres very well to hydrophilic surfaces, but the attachment force decreases with an increasing water contact angle. Although the egg cannot achieve attachment while submerged in water, it can reattach itself after dislodgement from a surface, making its adhesive mechanism temporary, and arguably long-term [77], depending on the conditions. This replicability of attachment can accomplish attachment site optimization to ensure suitable environmental conditions for embryonic development. This includes fixation in preferable environmental conditions, but also adjustment in case of environmental changes. The mechanism described herein copes with different degrees of surface roughness but is affected by the surface chemistry of the substrate. Other adhesive secretions in insects interestingly perform differently, although they serve a very similar function: the larval glue of the fly *Drosophila melanogaster* consists of glycosylated proteins and is used to anchor the pupa to different substrates [119]. In contrast to the egg glue of *P. philippinicum*, the glue of *D. melanogaster* larvae adheres well to various substrates independent of their surface chemistry or roughness [120]. This leads to the assumption that the egg deposition in *P. philippinicum* favors hydrophilic substrates and suggests preferable deposition site selection for the eggs in the natural habitat.

Knowledge about this mechanism can support ecologists and conservationists. Elucidating the nature of the attachment mechanism helps in understanding the dispersal as well as the life history of the species. This can help in quantifying fecundity for conservation purposes of the insect species [120]. Information on the attachment sites can help the conservation of plants and gauging the population density [1,121]. The details of this potential transitory state between non-adhesive and permanently attached eggs can be useful for evolutionary biologists. 

Furthermore, this repeatable and water-controlled mechanism can stimulate biomimetic research in the field of bioadhesives [48,122,123,124]. The origin and biochemical nature of the glue, however, remain elusive and should be subject to future studies.
